# Health Effects Due to Radionuclides Content of Solid Minerals within Port of Richards Bay, South Africa

**DOI:** 10.3390/ijerph13121180

**Published:** 2016-11-25

**Authors:** Felix B. Masok, Paulus L. Masiteng, Risimati D. Mavunda, Peane P. Maleka

**Affiliations:** 1 Applied Physics and Engineering Mathematics Department, University of Johannesburg, P.O Box 17011, Doornfontein 2028, South Africa; masokfelix@gmail.com (F.B.M.); plmasiteng@uj.ac.za (P.L.M.); 2 South African Nuclear Energy Corporation (Necsa), P.O Box 582, Pretoria 0001, South Africa; 3 Department of Nuclear Physics, iThemba LABS, National Research Foundation, P.O Box 722, Somerset West 7129, South Africa; pmaleka@tlabs.ac.za

**Keywords:** geogenic, activity concentration, hazard index, mineral samples, cancer risk

## Abstract

This study assessed the radiological health hazards to various body organs of workers working within Transnet Precinct in Richards Bay in Kwazulu-Natal, South Africa due to radionuclide content of mineral ores often stored within the facility. Thirty samples were collected from five mineral ores (rock phosphate, rutile, zircon, coal and hematite) and analyzed for ^238^U, ^234^U, ^226^Ra, ^210^Pb, ^235^U, ^232^Th, ^228^Ra, ^228^Th and ^40^K using delayed neutron activation analysis and low energy gamma spectroscopy. Rutile was found to be the most radioactive mineral ore within the facility with ^210^Pb concentration of 759.00 ± 106.00 Bq·kg^−1^. Effective annual dose rate in (mSv·y^−1^) delivered to different organs of the body: testes, bone marrow, whole body, lungs and ovaries from mineral ores were such that dose from mineral ores decreased in the order coal > rutile > rock phosphate > hematite > zircon. The organs with the highest received dose rate were the testes and this received dose was from coal. However, all of the calculated absorbed dose rates to organs of the body were below the maximum permissible safety limits.

## 1. Introduction

The exposure of human beings to naturally occurring radiation arises mainly from cosmic sources and terrestrial radioactive materials present in minerals and soils at different trace levels [1]. The science of radioactivity has been studied in great detail since its discovery in 1896. Radiation physics has been applied for the benefit of man in various fields such as medicine, biology, agriculture, industries and electric power generation [2]. As a result of application of radiation, humans can be exposed to radiation from different sources depending upon their activities and surroundings [3]. For instance, patients who are treated with medical irradiation or members of staff working in mining and nuclear industries may receive higher radiation exposure levels than members of the general public [4]. 

The National Council on Radiation Protection Measurement (NCPRM) pronounced ionizing radiation arising from radionuclides in the natural environment as the most obvious source of radiation to which all individuals are exposed (both in working and public environments) [5]. Hence, human exposure is inevitable. This radiation accounts for 85% of annual exposure dose received by the world population [6]. However, the International Atomic Energy Agency (IAEA) reported that exposure from natural radiation is of more concern to those working with mineral ores and naturally occurring radioactive material than the general public [7]. Nevertheless, any dose of radiation involves a possible risk to human health [6] from a health physics point of view. 

Millions of tons of mineral sands containing zircon, ilmenite and rutile, with xenotime and monazite are mined from many countries of the world [8]. These minerals are considered to be naturally-occurring Radioactive Materials (NORM) due to the presence of thorium and uranium in mineral grains [9]. In order to protect workers and the general public health against the radiological risk originating from naturally occurring radiation, concentrations of radionuclides and their biological effects on humans need to be assessed [7]. This has become the focus of greater attention by health scientists and the International Atomic Energy Agency (IAEA) in recent years [10]. Geological scientists are probing the earth’s crust to measure radiation levels so as to quantify the hazards and doses affecting people, animals, plants and all kinds of life [7]. In this study, we are contributing a small piece of information by investigating the anthropogenic radionuclide content of NORMs in some selected minerals available within the port of Richards Bay in the Kwazulu-Natal province of South Africa either for export or usage as raw materials by nearby companies. The aim is to determine the radiological health effects due to the concentration of naturally occurring radionuclides in these mineral samples, so as to inform management to implement safe working environments free of radiation hazards for those workers working with mineral ores within the Transnet facility.

## 2. Materials and Methods

### 2.1. Review of Richards Bay

**Figure 1:**
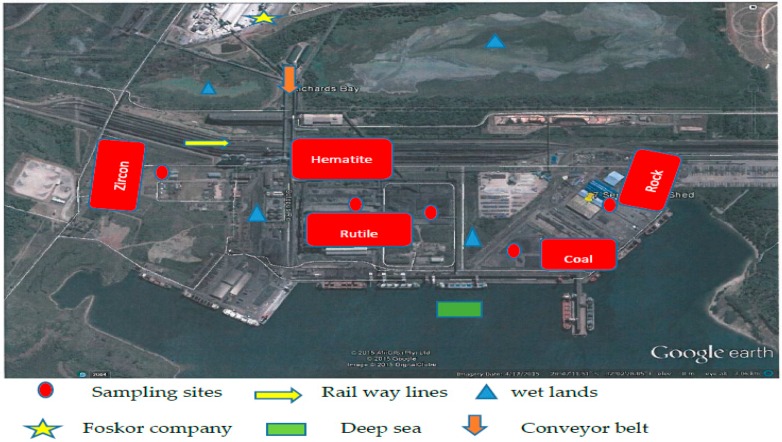
Arial view of the port in the Richards Bay area [14].

This study was conducted within the Richards Bay precinct located at latitude of 28.48′ S and a longitude of 32.02′ E approximately 199 km north of Durban on the east coast of South Africa. Commissioned in 1976, Port of Richards Bay is the largest in South Africa and handles about 40% of the country’s total port demand [11]. The port has stimulated the establishment of several industries in Richards Bay including Foskor, Richards Bay Coal Terminal (RBCT), and Richards Bay Minerals (RBM). About 95% of 2.0 million metric tons of heavy minerals including ilmenite, rutile and zircon mined by RBM are exported annually [12] via this port. Similarly, coal exportation by RBCT has been increasing annually up to 71.4 million metric ton in 2014 [13]. Rock phosphate is being mined and beneficiated in Limpopo province before it is transported by rail to Richards Bay and stored within the Transnet facility (see Figure 1). It is often conveyed through the conveyor belt (see Figure 1) from the open shade store at Richards Bay into the Foskor Company for the production of sulphuric acid (H_2_SO_4_), Phosphoric acid (P_2_O_5_) and granular fertilizer (MAP/DAP). The company exports about 90,000 metric tons of fertilizer annually [11]. The increasing demand for these minerals (rock phosphate fertilizer, coal, zircon, rutile and hematite) [11] has made supplying companies increase their availability within the port terminal almost daily throughout the year and provides over a thousand job opportunities to residents of the towns of Esikhawini, Empangeni, and Richards Bay. 

### 2.2. Sampling Techniques

In March 2016, a total of 30 samples were collected from five sampling sites within Richards Bay. At each sampling site, ten samples were collected within a distance of 1 m away from each other [15] at a depth of 5 cm using hand trowels into high density Ziploc polyethylene bags. The collected samples were thoroughly mixed for homogeneity out of which a representative sample [15] of about 1 kg was packaged and properly marked with unique sample identification codes before moving to the next sampling point. These procedures were repeated at each sampling point until six representative samples were collected from each sampling site. Thirty representative samples (six each from rock phosphate, coal, rutile, zircon and hematite) were finally collected and transported to Nuclear Energy Corporation of South African (Necsa) for preparation and analysis in the radio-analysis laboratory.

### 2.3. Sample Preparation

Samples were separately dried at 80 °C for 24 h. The 80 °C temperature was necessary because polonium is volatile at a higher temperature [15] from which lead-210 was measured while the 24 h were to ensure the samples attained a constant weight. Each sample was then milled into a fine powder using an electrical laboratory miller to allow for representative sub-sampling for various analysis techniques. Milled samples were packed into cylindrical plastic containers, which fit the geometry of the detector and were sealed using silicon sealant and left for five weeks (>7 half-lives of ^222^Rn and ^224^Ra) before counting. This was necessary to ensure the daughter products of ^226^Ra up to ^210^Pb and of ^228^Th up to ^208^Pb in order to attain secular equilibrium with their parent radionuclides ^238^U and ^232^Th, respectively [15,16]. 

### 2.4. Experimental Techniques

Gross alpha and beta measurements were performed as a first order estimate for total activities of each sample using a gas flow proportionality counter based on the Necsa adopted method [17]. Delayed neutron counting was utilized to determine ^235^U at a typical detection limit of 1.7 Bq·kg^−1^, while ^234^U was derived from ^238^U by applying natural abundance ratio ^238^U, and ^232^Th were analyzed using neutron activation analysis at detection limits of 120 Bq·kg^−1^ and 40 Bq·kg^−1^ for uranium-238 and thorium-232, respectively. High energy gamma spectroscopy was employed for the analysis of ^226^Ra, ^228^Ra and ^228^Th, while ^40^K and ^210^Pb were determined using low energy gamma spectroscopy. All analyses were carried out at Necsa, and the detailed analytical methods are fully documented in the Radio-Analysis Quality Management System, based on ISO/IEC 17025 Standards [17].

## 3. Radiological Health Hazards Assessment

All minerals and raw materials contain radionuclides of natural origin and are significantly radioactive up to 4000 Bq·kg^−1^ [7]. Most important for the purpose of radiation protection are the radionuclides in the ^238^U and ^232^Th decay series as well as ^40^K [6]. The gamma radiation from natural radionuclides and cosmic rays constitutes the external exposure, while those derived from inhalation and ingestion through food and drinking water constitute internal exposure to humans [7].

Upon exposure, the amount of energy deposited in living tissue is expressed in terms of a quantity “dose”. The radiation dose may come from any radionuclide, or a number of radionuclides as a result of decay of ^238^U and ^232^Th series. However, radiation absorbed doses depend on the intensity and energy of radiation, exposure time, the area exposed and the depth of the energy deposition [6]. In order to assess the health effects of nuclides to workers handling the minerals and other people living in close proximity, some dose quantities were evaluated. 

### 3.1. Absorbed Dose Rate Determination

This is the quantity of radiation energy absorbed per kilogram of tissue and expressed in units of Gray (Gy) [18]. The gamma absorbed dose rate is calculated using Equation (1) given by [1] and conversion factors of 0.462, 0.621, and 0.0417 nGy·h^−1^/Bq·kg^−1^ for ^226^Ra, ^232^Th and ^40^K assuming the contributions of ^137^Cr, ^90^Sr and ^235^U decay series to total dose from the environmental background is negligible [18]:

AD_rate_ (nGy·h^−1^) = 0.462 A_Ra_ + 0.621 A_Th_ + 0.0417 A_k_, (1)

where AD_rate_ is the absorbed dose rate in the air 1 m above the ground due to ^226^Ra, ^232^Th and ^40^K in mineral samples, A_Ra_, A_Th_ and A_k_ are the activity concentrations of ^226^Ra, ^232^Th and ^40^K in Bq·kg^−1^.

### 3.2. Radium Equivalent Dose Determination

Radioactivity in the environment depends on geological and geographical conditions and differs in mined minerals and soils of each region [1]. Therefore, the concentrations of ^226^Ra, ^232^Th and ^40^K in minerals are not uniform. Uniformity with respect to radiation exposure is defined in terms of radium equivalent activity (Ra_eq_) in Bq·kg^−1^. Radium equivalent activity (Ra_eq_), which is a single index used to describe the gamma output from different mixtures of nuclides (radium, thorium and potassium) in materials, is calculated from Equation (2) [19,20]:

Ra_eq_ (Bq·kg^−1^) = {A_Ra_} + {10/7} A_Th_ + {10/130} A_k_, (2)

where A_Ra_, A_Th_ and A_k_ are as defined in Equation (1) above. This calculation was done based on the assumption that 370 Bq·kg^−1^ of ^226^Ra from ^238^U, 259 Bq·kg^−1^ of ^232^Th and 4810 Bq·kg^−1^ of ^40^K have the same gamma ray dose rate with each radionuclide producing an effective dose of 1.5 mGy·y^−1^ [10].

### 3.3. Annual Effective Dose Determination

Amazingly, the same amount of doses from different types of radiation can produce dissimilar effects on human tissue. For instance, a dose from alpha particles can do much more damage than the same dosage amount from beta particles or gamma rays [17]. Therefore, the absorbed dosage in air cannot give the full representation of dose in a tissue. To compare absorbed doses of different types of radiation, they need to be weighted for their potential to cause certain types of biological damage [17]. This weighted dose is called the equivalent dose [17] and evaluated in units of Sieverts (Sv). Thus, in order to compare doses when different tissues and organs are irradiated, the equivalent doses to different parts of the body are also weighted given effective dose of radiation is more likely to cause cancer in the lungs than in the liver, and the reproductive organs are of particular concern because of the risk of hereditary effects [17]. The conversion factors (C-factors) for organ doses for some sensitive organs are presented in Table 1. 

However, estimating the annual effective dose rate due to natural radionuclides in these mineral samples required the consideration of factors such as (i) dose conversion coefficient of 0.7 Sv·Gy^−1^ [1] (quotient of effective dose rate and absorbed dose rate in air) that converts the absorbed dose rate in air to the effective dose; (ii) outdoor occupancy factor of 20% averaging 4.8 h spent working with mineral ores every day for a period of 1 year as proposed by [1]. Occupancy factor is the proportion of the total time during which an individual is exposed to radiation; (iii) 8760 h per year; and (iv) the conversion factor (10^−6^), which converts from nano (10^−9^) to milli (10^−3^). The annual effective dose is calculated using Equation (3) [19,22]:

AEDR (mSv·y^−1^) = [*D_rate_*(nGy·h^−1^) × *DCD* × *OF* × *T*] × 10^−6^, (3)

where AEDR is the annual effective dose rate, *D_rate_* is the effective absorbed dose rate in air *DCD* is the dose conversion factor (0.7), *OF* is the outdoor occupancy factor (0.2) and *T* is the time of the year in hours (8760 h)

### 3.4. Effective Dose Rate to Different Organs (D_organ_)

The quantity of absorbed radiation delivered to a particular organ of the body is calculated using the following relation [23]:

D_organ_ (mSv·y^−1^) = AEDR × C-factor, (4)

where AEDR is the annual effective dose rate and C-factor is the average organ conversion factor given in Table 1.

### 3.5. Radiological Hazard Indexes

For an insignificant radiation hazard, external and internal hazards indexes (H_ex_ and H_in_) should be less than 1 Bq·kg^−1^ [8]. They are determined from the mean activity concentrations of ^226^Ra, ^232^Th and ^40^K, using Equations (5) and (6) [24], respectively:

H_ex_ (Bq·kg^−1^) = [A_Ra_/370] + [A_Th_/259] + [A_k_/4810] < 1, (5)

H_in_ (Bq·kg^−1^) = [A_Ra_/185] + [A_Th_/259] + [A_k_/4810] < 1, (6)

where A_Ra_, A_Th_ and A_k_ are the activity concentrations of ^226^Ra, ^232^Th and ^40^K, respectively, and H_ex_ and H_in_ are external hazard index and internal hazard index, respectively.

### 3.6. Excess Lifetime Cancer Risk (ELCR) Determination

This is an estimation of the probability of acquiring cancer in a lifetime due to radiation exposure. *ELCR* is calculated using Equation (7) given by [23,25]:

*ELCR* (mSv·y^−1^) = AEDR × DL × RF, (7)

where DL is the life expectance factor averaging 62.45 years in South Africa [26]. RF is the risk factor (Sv), i.e., fatal cancer risk per Sievert, and the International Commission for Radiation Protection [27] uses a value of 0.05 (i.e., RF = 0.05).

## 4. Results

The average specific activity concentrations of ^238^U, ^234^U, ^226^Ra, ^235^U, ^210^Pb, ^232^Th, ^228^Ra, ^228^Th radionuclides as well as ^40^K from thirty representative samples (six each from rock phosphate, rutile, coal, zircon and hematite) are presented in Table 2. Furthermore, for the purpose of assessing health effects, the mean activity concentrations of ^238^U and ^232^Th decay series as well as ^40^K were calculated from Table 2, and the results obtained are presented in Table 3. The mean activity concentrations of ^226^Ra from ^238^U, and ^232^Th as well as ^40^K were used for the calculations of radiological hazard indices/indexes because they are considered highly radiotoxic natural radionuclides [26].

## 5. Discussion

### 5.1. Gross Alpha and Beta Concentrations in Rock Phosphate, Coal, Zircon, Rutile and Hematite

**Figure 2:**
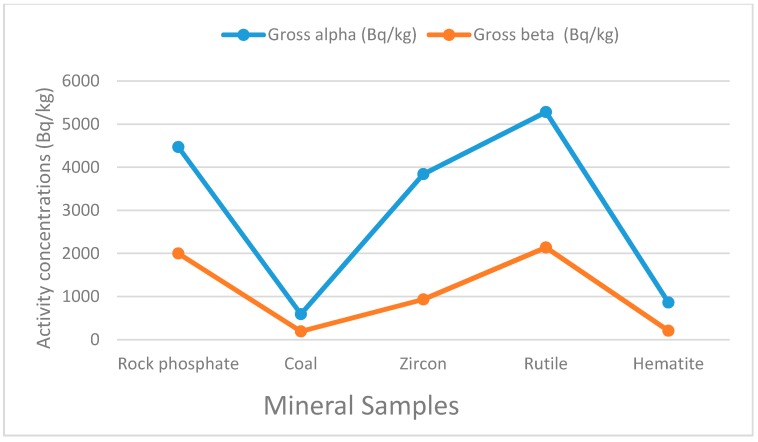
Gross alpha and beta activity concentrations in samples.

The gross alpha and beta activity concentrations are first order estimates that give the total radioactivity levels of the samples without regard to specific nuclides. In this study, rutile and coal were found to be the most and the least radioactive minerals, respectively, within the study area as shown in Figure 2. The former has gross alpha and gross beta activities of $5280.00\pm 430.00\;\mathrm{Bq}\cdot {\mathrm{Kg}}^{-1}\;\mathrm{and}\;2140\pm 50.00\;\mathrm{Bq}\cdot {\mathrm{kg}}^{-1},$ whereas the latter has the gross alpha and beta activities of $593.00\pm 179.00\;\mathrm{Bq}\cdot {\mathrm{kg}}^{-1}\;\mathrm{and}\;193.00\pm 20.00\;\mathrm{Bq}\cdot {\mathrm{kg}}^{-1}$ respectively (see Table 2). 

### 5.2. Average Activity Concentrations of Radionuclides in Mineral Ores in This Study Area

The mean activity concentrations of ^226^Ra and ^235^U content in the mineral samples analyzed range from 120.48 ± 8.35 Bq·kg^−1^ and 0.85 ± 0.06 Bq·kg^−1^ in coal to 468.50 ± 71.25 Bq·kg^−1^ and 22.30 ± 3.90 Bq·kg^−1^ in rutile. Similarly, the average activity of ^232^Th ranges from 72.00 ± 3.20 Bq·kg^−1^ in zircon to 809.27 ± 43.17 Bq·kg^−1^ in coal (see Table 3), whereas that of ^40^K ranges from 83.00 ± 39.00 Bq·kg^−1^ in rutile to 630.00 ± 0.01 Bq·kg^−1^ in coal (see Table 3). The errors in the activity concentrations were calculated using propagation of uncertainty equations based on the weighted average of the radionuclides and presented as plus or minus the measured activity of the sample. 

**Figure 3:**
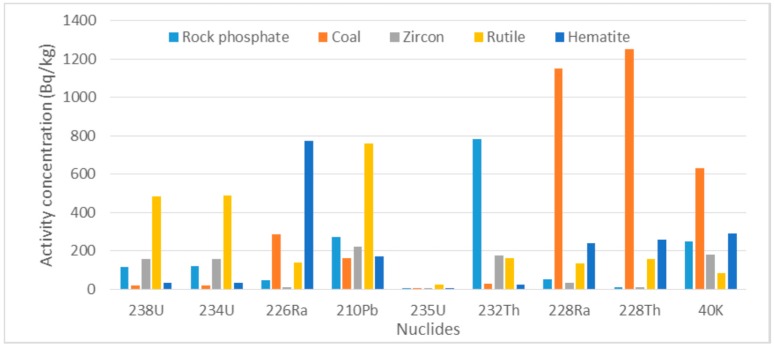
Concentrations of nuclides in various minerals within the Transnet facility in Richards Bay.

As shown in Figure 3 and Table 2, coal has more concentrations of ^228^Ra, ^228^Th, and ^40^K, whereas rutile has more concentrations of ^238^U, ^234^U, and ^210^Pb. The concentrations of ^40^K in rock phosphate, zircon, rutile and hematite are all lower than the world average of 420 Bq·kg^−1^ given by the International Atomic Energy Agency [6]. 

### 5.3. Comparison of Average Activity Concentrations of Radionuclides in Mineral Ore from This Study Area and Other Countries

The activity concentration of naturally occurring radioactive nuclides in rock phosphate, coal, rutile and zircon from this study area were compared with other countries of the world in Table 4, Table 5 and Table 6, respectively. In Table 4, rock phosphate from this study area displays relatively low concentrations of ^238^U and ^226^Ra, compared to many countries such as USA, Algeria, Morocco, Senegal, Tunisia, Egypt and Jordan. The low activity concentrations of these nuclides in rock phosphate make it is suitable for fertilizer production for the reason that soil contamination with uranium and its progeny will be lessened; hence human exposure from agricultural produce is minimized. This is a major inspiring factor for the high demand and export of rock phosphate and fertilizer from South Africa [11].

The concentrations of ^238^U, ^226^Ra and ^210^Pb, ^232^Th, ^228^Th and ^40^K in coal from this study area are higher than in many countries including USA, UK, Hungary (see Table 5). However, the International Atomic Energy Agency has pronounced coal with high mineral content (clay, quartz, pyrite and carbonate) as high quality minerals [8]. This may be the motivation for the increasing global demand for South African coal totaling 72.4 million tons in 2014 [13].

### 5.4. Assessment of Radiological Health Effects

The energy of ionizing radiation is high (>10^6^ eV) enough to liberate electrons from an atom, hence it can damage living tissue (IAEA 2013). Radiological health hazards (absorbed dose (AD_rate_), annual effective dose (AEDR), excess lifetime cancer risk (ELCR) and radiological hazard indexes) owing to the concentrations of radionuclides in these samples were calculated and are presented in Table 6.

The quantity of radiation absorbed by workers on site per kilogram of tissue (absorbed dose rate) due to radionuclides in these mineral samples were found to be 248.94, 584.48, 115.51, 3131.26 and 236.12 nGy·h^−1^ for rock phosphate, coal, zircon, rutile and hematite, respectively. Excess lifetime cancer risks of 0.95, 2.24, 0.44, 1.19 and 0.90 mSv·y^−1^ were obtained for rock phosphate, coal, zircon, rutile and hematite, respectively (see Table 6). These values are far below the value of 20 mSv·y^−1^ recommended by the International Commission on Radiological Protection (ICRP) for mine and radiation workers [26]. 

Furthermore, radium equivalent activity indices (Ra_eq_) were found to be 560.42, 1328.12, 254.36, 690.11 and 522.79 Bq·kg^−1^ for rock phosphate, coal, zircon, rutile and hematite, respectively. These values should be within the recommended value of 370 Bq·kg^−1^ for negligible health effects [26]. Coal has the highest radiological hazard indexes, though rutile was found to be more radioactive. This is because coal has a high concentration of nuclides from ^232^Th series and ^40^K compared to rutile with only high concentrations of ^226^Ra. 

### 5.5. Effective Dose Rate to Various Parts of the Body

Absorbed radiation dose rate and its effect on tissues depend on its intensity and energy, type of radiation, exposure time, organ exposed and the depth of the energy deposition [17]. The effective doses to various organs of the body were calculated from Equation (4) and presented in Table 7. The organs considered in this study are very sensitive to radiation [21]. The effective dose rate to all the organs investigated (testes, bone marrow, whole body, lungs and ovaries) were below the permissible limit, hence the tendency of long-term effect; cancer and hereditary is insignificant. 

**Figure 4:**
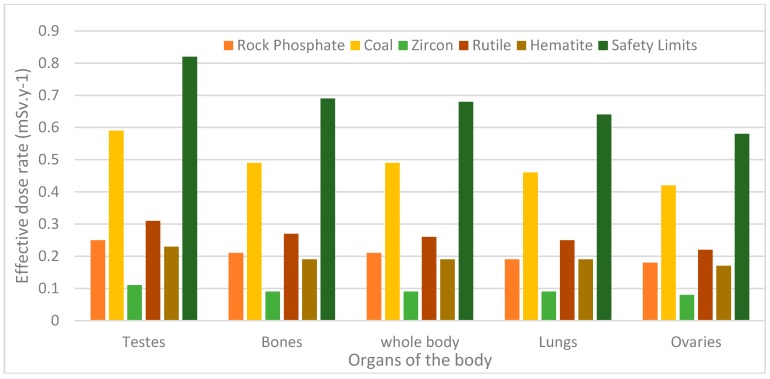
Annual effective dose rate delivered to selected organs of the body.

From Figure 4 and Table 7, coal is the highest contributor of annual effective dose rate to all the five body organs investigated. The least contributor of annual effective dose rate to the various body organs was found to be zircon, and this is attributed to the low concentration of nuclides within the sample.

The concentrations of ^238^U, ^234^U, ^226^Ra, ^210^Pb, ^235^U, ^232^Th, ^228^Ra, ^228^Th nuclides and ^40^K were not uniform in all of the samples. The disequilibrium in concentration of nuclides in these samples could be due to the nature of their parent rock, difference in the chemical and physical properties of the mineral, weathering which may cause emigration of some nuclides [27], and the fact that gases such as ^222^Rn (in ^238^U and ^226^Ra series) and ^220^Rn (^232^Th) escape from the sample.

## 6. Conclusions

From the experimental and computational work in this study, we can conclude that rutile is the most radioactive mineral within the Richards Bay precinct with the major contributing nuclide being ^210^Pb from ^238^U decay series. The concentrations of nuclides from the natural ^238^U decay series in rock phosphate is lower compared to most other countries in the world, hence this may result in less soil contamination when used for fertilizer production. The annual effective dose rates to human body organs (testes, whole body, lungs, ovaries and bone marrow) were below the maximum permissible limits, hence long term effects such as cancer and hereditary effects are improbable. 

Therefore, handling coal, rock phosphate, rutile, zircon and hematite for export or factory usage within Richards Bay at present poses negligible effects on workers, even on a long term basis. However, there is a need to sustain current safety methods such as immediate cleanup of spillage and proper ventilated working environment in order to avoid excessive accumulation of radiation dose. 

## Figures and Tables

**Table 1:** Average values of C-factors for different organs or tissues taken from [5,21].

Organ	C-Factor
Testes	8.2 × 10^−1^
Bone marrow	6.9 × 10^−1^
Whole body	0.68 × 10^−1^
Lungs	6.4 × 10^−1^
Ovaries	5.8 × 10^−1^

**Table 2:** Average concentrations of radionuclides in representative mineral samples.

NORM		Mean Activity Concentrations of Nuclides in Minerals (Bq·kg^−1^)				
Origin	Nuclides	Rock Phosphate	Coal	Zircon	Rutile	Hematite
^238^U Series	^238^U	118.00 ± 7.00	18.40 ± 1.20	158.00 ± 9.00	485.00 ± 84.00	31.50 ± 1.90
	^234^U	119.00 ± 7.00	18.50 ± 1.20	159.00 ± 9.00	489.00 ± 85.00	31.70 ± 1.90
	^226^Ra	44.50 ± 8.50	285.00 ± 31.00	11.00 ± 4.00	141.00 ± 10.00	772.00 ± 27.00
	^210^Pb	270.00 ± 1.00	160.00 ± 0.01	220.00 ± 0.01	759.00 ± 106.00	170.00 ± 0.01
^235^U Series	^235^U	5.45 ± 0.31	0.85 ± 0.06	7.28 ± 0.41	22.30 ± 3.90	1.45 ± 0.09
^232^Th series	^232^Th	783.00 ± 24.00	27.80 ± 1.50	174.00 ± 5.00	163.00 ± 6.00	23.90 ± 2.80
	^228^Ra	51.00 ± 1.00	1150 ± 80.00	32.00 ± 0.01	133.00 ± 16.00	239.00 ± 27.00
	^228^Th	11.00 ± 4.00	1250.00 ± 120	10.00 ± 4.60	155.00 ± 12.00	258.00 ± 42.00
Primordial	^40^K	240.00 ± 1.00	630.00 ± 0.01	180 ± 0.01	83.00 ± 39.00	290.00 ± 0.01
Gross alpha		4470.00 ± 940.00	593.00 ± 179.00	3840.00 ± 340	5280.00 ± 430.00	860.00 ± 192.00
Gross beta		2000.00 ± 120.00	193.00 ± 20.00	933.00 ± 36.00	2140 .00 ± 50.00	207.00 ± 20.00

Norm: Natural Occurring Radiation Materials.

**Table 3:** Mean activity concentration of ^226^Ra, ^232^Th decay series and ^40^K in rock phosphate, coal, zircon, rutile and hematite.

Minerals/NORMs	Mean Activity of Series (Bq·kg^−1^)			
	^226^Ra	^235^U	^232^Th	^40^K
Rock phosphate	137.63 ± 5.88	5.45 ± 0.31	281.67 ± 9.67	250.00 ± 0.01
Coal	120.48 ± 8.35	0.85 ± 0.06	809.27 ± 43.17	630.00 ± 0.01
Zircon	137.00 ± 5.50	7.28 ± 0.41	72.00 ± 3.20	180.00 ± 0.01
Rutile	468.50 ± 71.25	22.30 ± 3.90	150.33 ± 11.33	83.00 ± 39.00
Hematite	251.30 ± 7.70	1.45 ± 0.09	173.63 ± 23.93	290.00 ± 0.01

**Table 4:** Comparison of activity concentrations of ^238^U, ^232^Th and ^226^Ra in rock phosphate from these study with other countries.

Country	^238^U (Bq·kg^−1^)	^226^Ra (Bq·kg^−1^)	^232^Th (Bq·kg^−1^)
South Africa (^++^)	118	44.5	783
USA (Western) (^+,^*)	259–3700	1540	3.7–22
USA (Florida) (^+,^*)	1500–1900	1800	16–59
Brazil (^+,^*)	114–880	330–700	204–753
Chile (^+,^*)	40	40	30
Algeria (^+,^*)	1295	1150	56
Morocco (^+,^*)	1500–1700	1500–1700	10–200
Senegal (^+,^*)	1332	1370	67
Tunisia (^+,^*)	590	520	92
Egypt (^+,^*)	1520	1370	26
Jordan (^+,^*)	1300–1850	28–90	NA
Australia (^+,^*)	15–900	28–90	5–47

^+^ [10], * [6], ^++^ [present study], NA (not applicable).

**Table 5:** Comparison of activity concentrations of radionuclides in coal of this study with other countries.

Country	Activity Concentrations of Nuclides (Bq·kg^−1^)					
	^238^U	^226^Ra	^210^Pb	^232^Th	^228^Ra	^40^K
South Africa ^×^	18.4	285	160	27.80	1150	630
USA **	6–73	8.9–59	12–78	4–21	N^+^	N^+^
UK **	7–19	8–22	N^+^	7–19	N^+^	55–314
Hungary **	20–480	NA	N^+^	N^+^	12–97	30–384
China **	Range 10–25			Av. 25		
Greece **	111–390	44–206	59–205	N^+^	9–41	N^+^
Romania **	Av. 80.00	Av. 126	Av. 210	Av. 62	N^+^	N^+^

N^+^ (Not applicable), ^×^ (Present study), ** [10], Average (Av.).

**Table 6:** Calculated absorbed dose (AD_rate_), annual effective dose rate (AEDR), excess lifetime cancer risk (ELCR), and radiological hazard indexes (Ra_eq_, H_ex_, H_in_).

Samples	AD_rate_ (nGy·h^−1^)	AEDR (mSv·y^−1^)	ELCR (mSv·y^−1^)	Radiological Hazards Indexes (Bq·kg^−1^)		
				Ra_eq_	H_ex_	H_in_
Rock phosphate	248.93	298.12	0.95	560.42	1.51	1.88
Coal	584.48	711.77	2.24	1328.12	3.58	3.91
Zircon	115.51	134.05	0.44	254.36	0.69	1.06
Rutile	3131.26	358.09	1.19	690.11	1.86	3.13
Hematite	236.12	275.59	0.90	522.79	1.41	2.09

**Table 7:** Effective annual dose rate delivered to particular organs of the body.

Samples/Organs	Effective Dose Rate (mSv·y^−1^)				
	Testes	Bones	Whole	Lungs	Ovaries
Rock phosphate	0.25	0.21	0.21	0.19	0.18
Coal	0.59	0.49	0.49	0.46	0.42
Zircon	0.11	0.09	0.09	0.09	0.08
Rutile	0.31	0.27	0.26	0.25	0.22
Hematite	0.23	0.19	0.19	0.19	0.17
Permissible limit [21]	0.82	0.69	0.68	0.64	0.58
